# Outcome Reporting Bias in Randomized Clinical Trials: A Literature Review

**DOI:** 10.7759/cureus.119604

**Published:** 2026-11-13

**Authors:** Genavieve Prieto, Danielle Ucci, Samantha Asseraf, Edward Tobinick

**Affiliations:** 1 Neurology, Institute of Neurological Recovery, Boca Raton, USA

**Keywords:** outcome switching, randomized clinical trials (rcts), reporting bias, research transparency, selective outcome reporting

## Abstract

Randomized controlled trials (RCTs) are fundamental to evidence-based medicine; however, their reliability depends on the complete, accurate, and transparent reporting of study outcomes. Outcome reporting bias (ORB), including selective outcome reporting (SOR) and outcome switching, can distort interpretations of treatment efficacy and safety by selectively omitting, modifying, or emphasizing findings based on direction or statistical significance. This literature review examines the manifestations, prevalence, contributing factors, and consequences of ORB in RCTs, with particular emphasis on SOR and outcome switching. Evidence from systematic reviews and cross-sectional studies demonstrates that discrepancies between prospectively specified and published outcomes remain common, affecting approximately one-third of trials in broad assessments. Although such discrepancies do not necessarily indicate reporting bias, undisclosed or inadequately justified modifications undermine transparency and hinder independent evaluation of trial findings. Despite the widespread adoption of prospective trial registration and established reporting guidelines, persistent inconsistencies highlight the limitations of existing safeguards. Strengthening these practices is essential to improve the reliability of RCT evidence by reducing reporting bias and preserving the integrity of clinical research.

## Introduction and background

In clinical research, bias refers to systematic error that can distort the measurement, estimation, or interpretation of study findings, potentially leading to conclusions that deviate from the truth [1,2]. Unlike random error, which results from chance variation, systematic bias arises from aspects of research planning, design, conduct, analysis, or reporting. Randomized controlled trials (RCTs) are regarded as a principal method for evaluating the efficacy of health care interventions and are central to drug development because they help avoid systematic bias [1,3-5]. Randomization is specifically intended to reduce selection bias by distributing known and unknown prognostic factors between treatment groups [1,5]. However, an RCT may achieve appropriate randomization while remaining vulnerable to biases arising before, during, or after the trial itself [2,6-8]. The protection afforded by randomization depends on appropriate implementation and adequate allocation concealment, yet it does not eliminate the risk of other biases throughout the research lifecycle [1,2,5].

Potential sources of bias can arise at the earliest stages of clinical research, even before participants are enrolled. The choice of research question, study objectives, trial design, interventions, comparator groups, and outcomes may be influenced by scientific priorities, available funding, commercial interests, regulatory considerations, investigator preferences, or competing academic interests [9]. These design biases often occur when trial parameters are determined before the decision to initiate the RCT [10]. These influences can affect which hypotheses are investigated and how they are evaluated. Research agendas may also reflect financial or institutional priorities rather than the most pressing clinical questions [8,9,11]. Although such influences do not necessarily indicate the presence of systematic bias, they can create conditions in which particular research questions, interventions, or findings receive disproportionate attention. Prospective trial registration provides an early safeguard by publicly documenting core design choices and intended outcomes before participant enrollment, making subsequent changes easier to identify, though it cannot eliminate bias already present in the initial trial design.

Bias may also arise during participant selection, treatment allocation, and trial conduct. Selection and allocation bias can occur when randomization procedures are inadequate, or allocation concealment is compromised, allowing systematic differences between treatment groups [1,2]. Restrictive eligibility criteria and selective recruitment practices may produce trial populations that differ systematically from the broader patient population, thereby limiting the external validity and generalizability of trial findings [12]. During trial conduct, performance bias, loss of blinding, differences in intervention delivery, treatment adherence, co-interventions, or deviations from intended treatment protocols may influence observed outcomes [1].

Detection or ascertainment bias may arise when outcome assessments differ between groups or when investigators’ knowledge of treatment allocation influences measurement or interpretation [1,13]. Additional threats include attrition bias associated with missing outcome data, inappropriate participant exclusions, and analytical decisions that can alter estimated treatment effects [1,13]. Collectively, these examples show that bias is not confined to any single stage of an RCT but may arise through multiple methodological and operational processes, potentially affecting both the internal validity and generalizability of its findings [1,2,7,14].

A fundamental component of RCTs is the dissemination of trial findings, which enables scientific evidence to inform clinical practice, healthcare policy, and future research. However, this process is vulnerable to reporting bias, which occurs when the nature, direction, magnitude, or statistical significance of study results influences whether or how findings are disseminated [15-17]. Reporting bias can operate at the level of an entire study through publication bias, in which the likelihood of publication depends on the study’s findings, or within individual studies through selective reporting of outcomes and results [17,18]. Such practices may favor positive or statistically significant findings while minimizing, omitting, or withholding negative, null, or otherwise unfavorable results. Unlike biases arising from treatment allocation, intervention delivery, or outcome measurement, reporting bias can distort how findings are represented after data collection and analysis, influencing which results ultimately become part of the publicly available evidence base [18,19].

The evidence available to clinicians, researchers, and policymakers may not fully reflect the findings obtained during the investigation, potentially misrepresenting an intervention’s efficacy or safety [3,4,20-23]. These concerns may be further compounded by a research environment that places greater value on novelty and positive findings, contributing to the underreporting of negative, null, or confirmatory results [9,15-17]. Therefore, even when earlier stages of an RCT have been conducted rigorously, reporting bias can compromise the accuracy and completeness of the final published account and undermine the reliable communication of treatment effects [17-19].

Reporting bias encompasses multiple forms of selective dissemination and presentation of research findings, each of which can affect the completeness and accuracy of the published evidence. One form of reporting bias, outcome reporting bias (ORB), refers specifically to the selection of a subset of measured or analyzed outcomes for reporting based on their results. Selective outcome reporting (SOR), a closely related and often overlapping term, encompasses practices such as omitting outcomes, incompletely reporting results, selectively presenting analyses, or emphasizing particular outcome measures or assessment time points [18,21]. These practices may result in the preferential presentation of statistically significant or favorable results while minimizing or excluding nonsignificant, unfavorable, or otherwise less desirable findings [18,20].

One subset of SOR is outcome switching, also known as endpoint switching, in which outcomes reported in the final publication differ from those prospectively specified in the trial protocol or registration [20,24]. Outcome switching may involve introducing new outcomes, omitting prespecified outcomes, promoting secondary outcomes to primary status, downgrading primary outcomes, or modifying outcome definitions or assessment time points [18,21,24]. Although outcome modifications may be scientifically justified, undisclosed changes influenced by knowledge of the results can introduce reporting bias and undermine the reliability of the published conclusions [21,25]. Importantly, a discrepancy between prespecified and reported outcomes does not by itself establish bias; its significance depends on the timing, justification, transparency, and relationship of the modification to the observed results [20,25].

Because clinical decisions and guidelines rely on the publicly available evidence base, ORB and SOR can distort perceptions of treatment benefits and harms, leading to overestimation or underestimation of intervention effects and potentially misleading conclusions about efficacy or safety [14,18,26]. Selective reporting may alter the apparent balance between therapeutic benefits and adverse effects, obscure clinically meaningful findings, or create an incomplete picture of an intervention’s overall effectiveness [18,26]. These distortions extend beyond individual trials because published outcomes frequently contribute to systematic reviews, meta-analyses, clinical practice guidelines, regulatory evaluations, and subsequent research priorities [14,26]. When selectively reported findings are incorporated into evidence synthesis, the resulting estimates may no longer accurately reflect the complete body of available evidence [18,26,27].

Complete, accurate, and transparent reporting of clinical trial outcomes is therefore essential for maintaining research integrity, preserving the validity and credibility of published findings, and supporting reliable evidence-based decision-making [17,19,28]. Against this background, the present literature review examines reporting bias in RCTs, with particular emphasis on SOR and outcome switching, also known as endpoint switching. These practices were selected for focused examination because, despite increasing recognition in the literature, they remain insufficiently addressed in routine trial reporting and editorial oversight, which warrants greater attention to their prevalence, manifestations, and implications for research integrity [18,20,25]. By examining these issues within the broader context of bias throughout the clinical research lifecycle, this review aims to emphasize the importance of comprehensive and faithful outcome reporting in safeguarding scientific integrity, maintaining confidence in clinical research, and ensuring that treatment decisions are informed by an accurate and balanced representation of the available evidence [17,25,28].

## Review

Methods

This literature review was developed using references identified through non-systematic searches conducted in September 2026. Searches were performed in PubMed, PubMed Central, and Google Scholar to identify literature on bias in RCTs, selective reporting, outcome switching, endpoint switching, and discrepancies between trial protocols, registries, statistical analysis plans, regulatory documents, and published reports from 2000 to 2026. The literature considered included systematic and narrative reviews, meta-analyses, meta-epidemiological studies, cohort studies, cross-sectional studies, retrospective analyses, and case studies. Titles, abstracts, and full texts were reviewed to assess the relevance of publications to the review. Publications were selected or excluded based on their relevance to the identification, classification, prevalence, and implications of reporting bias, with particular emphasis on SOR and outcome switching. Earlier foundational publications were also considered when directly relevant, and publications unrelated to these methodological issues were excluded.

Reporting bias in RCTs

Reporting bias is a concept encompassing situations in which the nature of research findings influences whether or how those findings become publicly available. Two particularly important forms are study publication bias and ORB. In the former, completed trials with negative or null findings may remain unpublished; in the latter, the trial is published, but particular outcomes or analyses are selectively omitted, modified, or emphasized [29]. ORB therefore differs conceptually from complete nonpublication. A 2018 study characterized ORB as a missing-data problem arising when trialists select outcome results for publication after the results are known [30]. Although publication bias and ORB are among the most widely recognized forms, reporting bias can occur at multiple stages of dissemination and may also involve selective analysis, incomplete outcome reporting, preferential reporting of harms, or misleading emphasis on selected findings [11,19,20,29-31]. These related forms of reporting bias in RCTs are summarized in Figure 1.

**Figure 1:**
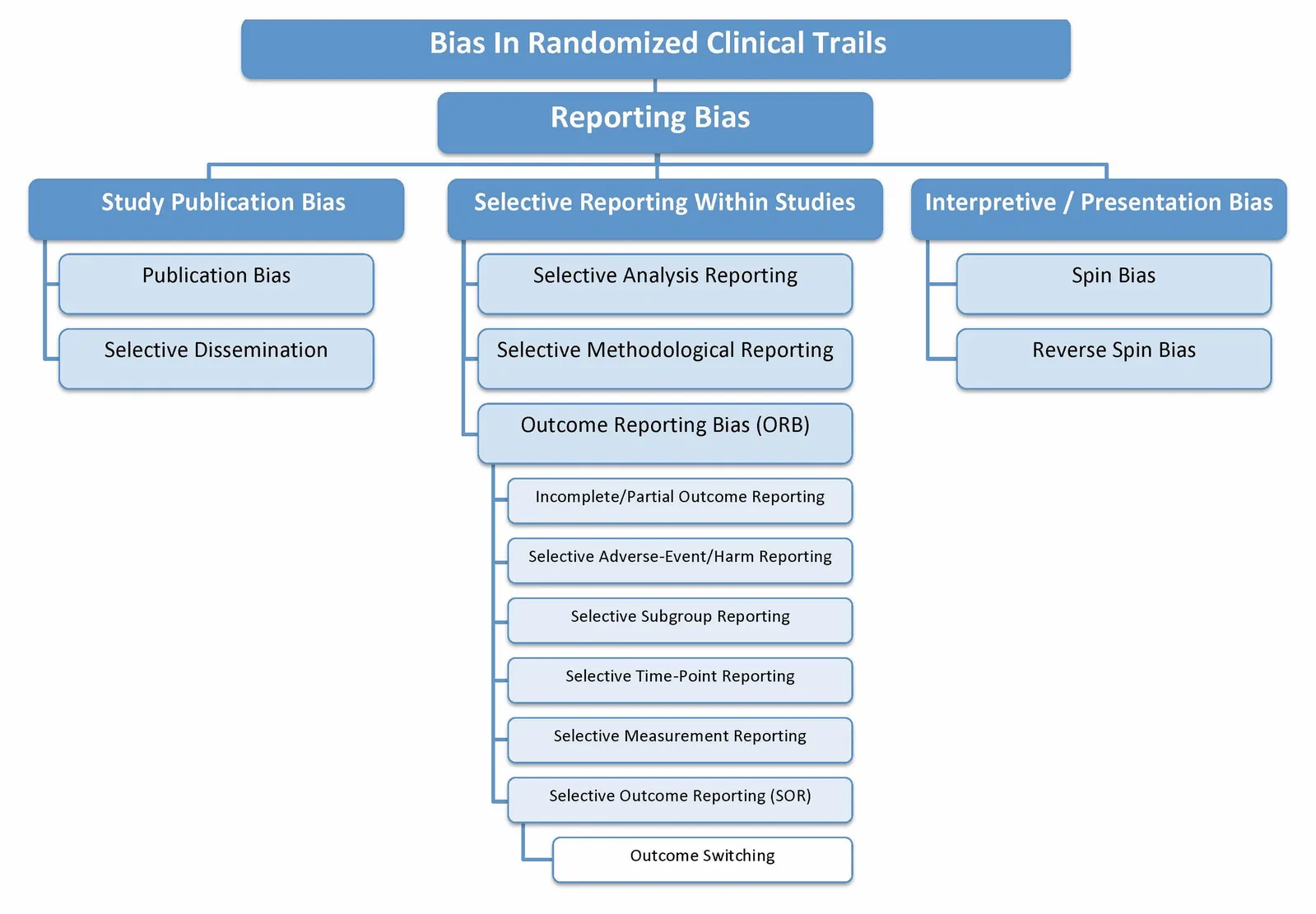
Conceptual taxonomy of reporting bias in randomized clinical trials Diagram illustrating major categories and subtypes of reporting bias in randomized clinical trials, including study publication bias, selective reporting within studies, and interpretive or presentation bias. Categories may overlap, and the taxonomy is illustrative rather than exhaustive or universally standardized [10,11,14,18-21,24,29-44]

The medical literature subsequently used for guidelines and systematic reviews may represent neither all completed trials nor all measured outcomes from published trials [29,30,45]. As a result, the published evidence base may differ systematically from the complete body of research that was actually conducted, limiting the ability of clinicians and reviewers to evaluate treatment effects using the full available evidence. Although reporting bias encompasses several distinct practices, this review focuses specifically on SOR and its subset, outcome switching, in RCTs. SOR involves the selective reporting, omission, or modification of trial outcomes, whereas outcome switching refers to changes in prespecified outcomes, including their introduction, removal, or reclassification. Outcome switching may contribute to ORB when such changes are influenced by study results rather than being scientifically justified and transparently disclosed [18,21,30].

One classification used to describe discrepancies between registered and published primary outcomes comprises five types and is summarized in Table 1. Type I refers to a registered primary outcome reported as secondary; Type II refers to a registered primary outcome omitted from the publication; Type III refers to a new primary outcome introduced in the publication; Type IV refers to a published primary outcome that had been registered as secondary; and Type V refers to a difference in the timing of assessment [46]. Accordingly, Type III should not be used as a synonym for all ORB or all outcome switching. It specifically denotes the introduction of a new primary outcome under this classification [46]. This distinction becomes particularly important when evaluating trials in which the nominal outcome remains the same, but its operational definition is subsequently modified. In addition to the five types of registration-publication primary outcome discrepancies in RCTs, there are other types of discrepancies involving changes to important trial characteristics, such as the outcome definition, measurement variable or instrument, analysis metric, method of aggregation, partial or incomplete reporting, and selective reporting of time points or subsets. These additional discrepancies are also summarized in Table 1.

**Table 1:** Mechanisms of bias Table summarizing common forms of SOR and outcome switching identified through comparisons of trial registrations, protocols, and final publications. The Type I-V designations follow the classification of inconsistencies between registered and published primary outcomes. Categories identify reporting discrepancies and do not independently establish that the changes were influenced by study results SOR: selective outcome reporting

Discrepancy	Description	Example of how it appears	Source
Downgrading (Type I)	A registered primary outcome is subsequently reported as secondary or unspecified	A registered primary outcome is reported as a secondary outcome in the final publication	[14,46]
Omission (Type II)	A prospectively specified primary outcome is omitted from the final publication	A prospectively specified primary outcome is absent from the final publication	[18,20,31,46]
Introduction of new primary outcome (Type III)	A new or materially modified primary outcome, not specified as such in the registry, is introduced in the final publication	The registered primary endpoint is replaced with a newly defined primary endpoint that differs from the original specification	[46]
Upgrading (Type IV)	An outcome prospectively designated as secondary is subsequently reported as a primary outcome in the final publication	A registered secondary outcome is promoted to primary status	[14,46]
Changed assessment time (Type V)	The same outcome is assessed/reported at a different time than prospectively specified	A primary outcome specified for assessment at 24 hours is instead reported at 1 hour	[31,46]
Changed outcome definition	Criteria defining the endpoint are altered while the general outcome label may remain the same	Different responder criteria applied to the same instrument	[21]
Changed measurement variable/instrument	A different variable or measurement instrument is used to operationalize the outcome	Different questionnaire, domain, scale, or variable	[21,33]
Changed analysis metric	Changed analysis metric	The endpoint uses a different analytical expression	[33]
Changed method of aggregation	The statistic summarizing participant outcomes is changed	Mean outcome versus proportion of responders	[33]
Partial/incomplete reporting	An outcome is mentioned, but insufficient data are supplied for full interpretation or meta-analysis	P value without effect estimate/precision	[26,30,32]
Selective reporting of time points/subsets	Only selected measurements from several assessed time points or subsets are presented	Favorable time point emphasized while another is omitted	[18,26]

Selective outcome reporting

SOR occurs when the published report does not faithfully represent the outcomes that were prospectively specified, measured, or analyzed. It may involve omitting unfavorable or nonsignificant outcomes, introducing new outcomes after data have been collected, reporting only a subset of analyzed data, failing to provide results for measured outcomes such as adverse events, or changing the hierarchical status of an outcome [18].

The potential influence of SOR on the interpretation of treatment effects was illustrated by a 2018 case study that reanalyzed data from a randomized trial using eight alternative definitions of a bleeding outcome [21]. Depending on the outcome definition applied, estimated odds ratios ranged from 0.23 to 0.94, with corresponding p-values ranging from less than 0.001 to 0.89 [21]. These findings demonstrated that alternative definitions of the same clinical outcome could produce substantially different statistical conclusions. Although this analysis did not establish that selective reporting had occurred in the original trial, it illustrated how the choice of outcome definition and the selection of results for presentation could materially influence perceptions of treatment effectiveness [21]. Similarly, a 2019 cross-sectional study examined 389 RCTs and found that 33.4% had at least one change in the registered primary outcome [27]. Trials with primary outcome changes were associated with larger reported intervention effects than trials without such changes, providing further evidence of the potential consequences of discrepancies between prespecified and published outcomes [27].

Discrepancies between registration and publication are also particularly concerning when deviations are not disclosed [25,47]. Contemporary research further highlights the relevance of selective reporting. A 2024 cross-sectional study categorized selective reporting in trials into omission-type reporting (downgrading, omission, or ambiguous reporting) and addition-type reporting (upgrading, adding outcomes, or altering their measurement scope) [14], illustrating that selective reporting can affect not only whether an outcome appears in the final publication, but also the prominence and manner in which it is ultimately presented. Importantly, SOR is not limited to changes in which outcomes are reported. It may also involve selectively emphasizing or minimizing particular findings, thereby influencing the interpretation of a trial's results without necessarily changing its formally designated endpoints.

Evidence suggests that SOR is frequently associated with the direction and statistical significance of trial findings. In a 2009 study, 19 of 23 (82.6%) assessable primary-outcome discrepancies favored statistically significant results [31]. Similarly, empirical evidence indicates that statistically significant outcomes are more likely to be completely reported than nonsignificant outcomes, with reported odds ratios ranging from 2.2 to 4.7 [30]. Among 180 RCTs published in high-impact journals, major discrepancies were identified in 180 outcome instances, including omitted or demoted registered primary outcomes, introduction or upgrading of primary outcomes, and changes in assessment timing. Of the 82 major discrepancies for which P values were available, 54 (66%) favored statistically significant results [48].

More recent evidence from stroke trials further demonstrates the relationship between reporting patterns and outcome significance. A 2024 cross-sectional study found that negative findings were strongly associated with omission-type reporting, whereas positive findings were associated with addition-type reporting [14]. Together, these findings suggest that SOR can systematically distort the published evidence by influencing which results receive prominence, are fully reported, or are omitted altogether.

Outcome switching

A related but distinct methodological concern is outcome switching, also referred to as endpoint switching, which involves discrepancies between prospectively specified outcomes and those subsequently reported in a trial publication. It can include the omission of a registered primary outcome, the introduction of an unregistered primary outcome, the demotion of a primary outcome to secondary status, the promotion of a secondary outcome to primary status, or a change in the assessment time point [20,31,49].

Outcome switching has been repeatedly documented across clinical specialties. A 2009 study found discrepancies between registered and published primary outcomes in 46 of 147 adequately registered trials (31.3%) [31]. Similarly, a 2017 cross-sectional analysis identified outcome switching in 39 of 216 (18%) oncology RCTs [24]. A systematic review of 27 studies reported a median primary-outcome discrepancy rate of 31%, increasing to 41% among studies specifically comparing prospectively registered outcomes with published outcomes [20].

Subsequent systematic reviews have reinforced these findings. A 2018 review of 37 surveys and systematic reviews identified outcome-reporting inconsistencies ranging from 14% to 100%, reflecting substantial heterogeneity across biomedical research [19]. More recently, a 2023 systematic review and meta-analysis encompassing 89 articles estimated that approximately one-third of studies had at least one primary-outcome discrepancy (95% CI: 29%-37%), while secondary-outcome discrepancies were more prevalent (95% CI: 50%-75%) [25]. Collectively, these findings demonstrate that discrepancies between prospectively specified and published outcomes remain common despite the increasing adoption of trial registration.

Outcome switching may also be less obvious than replacing one named endpoint with another. An endpoint can retain the same general label while its underlying operational definition changes. Changes in the outcome definition, measurement instrument, method of assessment, or assessment time point can generate different trial results [21]. More recent approaches additionally distinguish components such as the measurement variable, analysis metric, method of aggregation, and time point [33]. Accordingly, determining whether an endpoint has changed requires comparing how the outcome was actually defined and analyzed rather than relying on a comparison of outcome names alone. Methodological approaches have similarly emphasized that incomplete outcome definitions may remain compatible with multiple possible results, limiting the ability of prospective registration to constrain subsequent reporting flexibility [50].

Even when an outcome has been registered, failure to specify its measurement variable, instrument, threshold, assessment method, analysis metric, aggregation rule, or assessment time point may permit multiple operational definitions of the same clinical concept. Such flexibility can produce different treatment-effect estimates and conclusions regarding statistical significance without an apparent change in the endpoint's name [21,33]. The principal forms of SOR and outcome switching, including changes in outcome hierarchy, definition, timing, measurement, and reporting completeness, are summarized in Table 2.

**Table 2:** Types of outcome reporting bias and reporting practices relevant to RCTs Table summarizing major forms of ORB that may affect RCTs and the evidence derived from them. The categories are not mutually exclusive, may coexist within the same trial, and not all ORBs and reporting practices relevant to RCTs are listed RCTs: randomized controlled trials; ORB: outcome reporting bias; SOR: selective outcome reporting

Type of reporting problem	Definition/description	Potential effect	Source
ORB	Reporting outcomes are influenced by the nature or significance of results	Distorts estimates of treatment efficacy or harm, potentially misleading clinical decision-making, systematic reviews, and meta-analyses	[29,30]
SOR	Selective omission, incomplete reporting, or modifying prespecified outcomes	Incomplete or unbalanced representation of trial findings, potentially exaggerating or underestimating treatment benefits and harms	[18,29,39]
Outcome/endpoint switching	The primary outcome reported in the publication differs from that prospectively specified, including omission, introduction, upgrading/downgrading, or altered timing	Weakens the distinction between prespecified confirmatory findings and post hoc findings	[20,24,31]
Incomplete/partial outcome reporting	An outcome is reported without enough quantitative information for interpretation or evidence synthesis	Prevents inclusion in meta-analysis and can selectively obscure unfavorable outcomes	[30,32]
Selective subgroup reporting	Highlighting favorable subgroups while omitting unfavorable subgroups, or selectively presenting subgroup comparisons	May suggest benefit in a subset that is not supported by the overall result, or conceal subgroup harms	[19,37]
Selective time-point reporting	Emphasizing favorable assessment times while omitting or minimizing less favorable findings at other times	May exaggerate or underestimate treatment effects by emphasizing selected follow-up times rather than the full prespecified pattern of results	[14,21,31]
Selective statistical significance reporting	Emphasizing significant findings while minimizing, incompletely reporting, or omitting other findings	May exaggerate apparent treatment efficacy and obscure null or unfavorable findings, leading to distorted conclusions and biased evidence synthesis	[14,18]
Selective measurement reporting	Reporting only certain scales, components, thresholds, or measurement methods from multiple available options	Can favor a particular treatment effect by concealing results from alternative measures or definitions, weakening comparability and reproducibility	[21,33]
Selective adverse-event/harm reporting	Harms measured during a study are omitted or incompletely reported	Treatment benefits can appear more favorable when adverse effects are incompletely represented	[11,36,41]

Although outcome switching may contribute to ORB, the presence of a discrepancy alone does not establish bias or improper conduct. Legitimate methodological changes can occur during a trial, and distinguishing justified modifications from potentially biased reporting requires sufficiently detailed prospective outcome specification, preservation of the original trial record, transparent disclosure of amendments, an explanation of their rationale, and documentation of the timing of these changes [25,34]. For changes to statistical analysis plans, investigators should identify the original and revised analyses, explain the reasons and timing of the changes, and disclose whether the modifications were made before or after access to treatment-group results.

An undisclosed deviation does not automatically establish that a change was motivated by the observed results; such a determination requires additional evidence concerning the timing, decision-making, and availability of alternative results. Accordingly, changes to prespecified outcomes should be transparently documented and explained so that readers can evaluate their implications for the trial's validity and interpretation [34,49]. These modifications, together with related forms of reporting problems and their potential effects on the interpretation of RCTs, are summarized in Table 2 and Table 3.

**Table 3:** Types of reporting bias and reporting practices relevant to RCTs Table summarizing major forms of reporting bias that may affect RCTs and the evidence derived from them. Reporting bias may occur at the study level, such as publication bias, or within a published trial through selective reporting, modification, omission, or emphasis of particular outcomes or analyses. The categories are not mutually exclusive, may coexist within the same trial, and not all reporting bias and reporting practices relevant to RCTs are listed RCTs: randomized controlled trials

Type of reporting problem	Description	Example of how it appears	Source
Study publication bias	Entire trials, particularly those with negative or null findings, are not published	The publicly available evidence disproportionately represents favorable studies	[10,29,32,35,39,43]
Spin or misleading emphasis	Results are presented in a more favorable manner than the primary findings warrant	Readers may perceive benefit despite neutral or unfavorable principal findings	[11,42]
Reverse spin bias	Narrative discounting or dismissal of statistically significant findings	Potentially effective treatments may be neglected or dismissed	[40]
Selective methodological reporting	Omitting material details about trial conduct, eligibility, protocol deviations, or analytical decisions that readers need to evaluate validity	Prevents independent assessment of trial validity, protocol fidelity, reproducibility, and the risk of bias in the reported results	[37,38]
Selective dissemination	Presenting different or selectively emphasized findings in abstracts, conference presentations, press releases, or public communications	May create a misleading public account of trial findings, distort perceptions of treatment efficacy or safety, and propagate selective interpretations beyond the full report	[11,44]
Selective analysis reporting	A preferred analysis is reported from multiple possible analyses or analytic specifications	Analytical flexibility may alter statistical significance or estimated effects	[19]

Consequences for trial credibility and evidence synthesis

Inconsistency between planned and reported primary outcomes weakens the ability to determine whether a trial's headline result reflects its original hypothesis or an outcome selected after multiple results became available. Such inconsistencies increase the possibility that chance or selective reporting, rather than a true treatment difference, accounts for the reported primary result [20]. Bias has been described as a systematic inconsistency capable of contaminating the primary comparison, making the recognition of potential sources of bias essential when evaluating a study's internal validity [51]. This uncertainty can undermine confidence in the internal validity of a trial because the evidentiary meaning of a primary endpoint depends, in part, on its having been defined before the comparative results are known. At the broader literature level, reporting bias also threatens the completeness and validity of the accessible evidence base because selective dissemination determines which findings are available to clinicians, systematic reviewers, and guideline developers [15].

Several factors may contribute to these reporting practices, including publication pressures, academic incentives, financial interests, and preferences for statistically significant or favorable findings. A 2022 study identified perceived journal preferences for more “exciting” results and financial incentives to disseminate favorable findings as potential contributors to reporting bias [29]. More broadly, academic reward systems that prioritize novelty and positive results may encourage preferential dissemination, while negative, null, or confirmatory findings receive less attention [15]. These pressures may be reinforced when authors and journals prioritize positive findings irrespective of whether they correspond to prespecified primary outcomes [52]. Empirical evidence is consistent with these concerns. A cross-sectional analysis of 13,238 completed interventional trials registered on ClinicalTrials.gov found that primary-outcome modifications made after trial completion and funding source were associated with the reporting of statistically significant primary outcomes [53]. Although these associations do not establish investigators' motivations or demonstrate causation, they underscore how research and publication incentives may contribute to discrepancies between prospectively planned and published outcomes.

A 2018 case study showed that varying only the definition of the endpoint could transform a result from essentially no evidence of benefit to strong statistical evidence of benefit [21]. Outcome switching or redefinition can affect not only how results are presented but also the apparent magnitude and statistical interpretation of the treatment effect itself. These trial-level distortions can then extend into the broader evidence base. SOR can propagate from an individual RCT into systematic reviews because outcomes that are missing or incompletely reported may not contribute to pooled analyses. If the probability of complete reporting depends on statistical significance, the available dataset for meta-analysis may systematically favor larger or more positive treatment effects [18,30]. A 2022 article reported that adjustment for suspected SOR reduced estimated treatment effects by at least 20% in 23% of reviewed meta-analyses. Among 42 meta-analyses initially reporting statistically significant findings, eight became nonsignificant after adjustment [18]. Thus, selective reporting within individual trials can alter the conclusions of evidence synthesis and, in some cases, the apparent statistical significance of pooled findings.

The downstream implications are therefore clinical as well as methodological. Reporting bias may ultimately affect clinicians and patients because treatment decisions, practice guidelines, and health policy depend on the published evidence base. An incomplete or selectively favorable literature can alter perceptions of a treatment's benefit-risk profile and undermine evidence-based decision-making [18,29]. When such distortions are incorporated into guidelines or policy, the effects of selective reporting may extend well beyond the original study population. Discrepancies may also extend beyond whether an outcome is reported to include differences in the numerical results and adverse events reported across sources. In a comparison of 110 trials with results posted on ClinicalTrials.gov and corresponding peer-reviewed publications, 15% described the primary outcome inconsistently and 20% reported inconsistent primary-outcome values. Serious adverse-event reporting also differed frequently; among trials with discrepant serious adverse-event counts, ClinicalTrials.gov reported more events than the corresponding publication in 33 of 38 cases [36].

SOR also occurs within a broader context of methodological deficiencies in trial outcome specification and analysis. In a survey of 161 randomized trials across several common clinical conditions, deficiencies included failure to clearly define primary outcomes, inappropriate subgroup analyses, and inadequate handling of missing data, illustrating how outcome-reporting concerns can coexist with other threats to trial validity [22].

These concerns are particularly relevant because RCT findings frequently inform treatment guidelines in areas with substantial morbidity and limited therapeutic options. A 2019 cross-sectional retrospective analysis examined RCTs and found that, on average, only approximately half of the Consolidated Standards of Reporting Trials (CONSORT) criteria were fulfilled. Completely defined prespecified primary and secondary outcomes were reported in 53.85% of the included trials [38]. The authors emphasized that inadequate planning or reporting can hamper the interpretation of RCT findings and that greater adherence to CONSORT could improve research quality [38]. Another retrospective analysis examining RCTs found that 22 out of 72 studies (31%) reported fewer than half of the CONSORT checklist items [54].

More recent studies suggest that SOR remains common despite the widespread use of trial registration. A 2024 cross-sectional study evaluated 82 registered RCTs published between 2020 and 2022 and identified selective reporting in 72 trials (87.8%) and in 497 of 819 outcomes (60.7%) [14]. Furthermore, omission-type selective reporting was associated with negative findings, whereas addition-type reporting was associated with positive findings, indicating that the discrepancies were related to the direction of study results [14].

Not all investigations have found a high prevalence of ORB when adequate prospective documentation was available. In a systematic review of 600 RCTs included in Cochrane reviews, the trial registry record or protocols for only 123 trials were identified (21%). Among those assessable trials, 110 (89%) were judged to have no risk of primary-ORB, seven (6%) were at low risk, and six (5%) were at high risk. The authors therefore found a comparatively low prevalence of primary-outcome ORB among adequately documented trials, while emphasizing the concerning inability to locate a registry record or protocol for most of the sampled RCTs [55]. These apparently differing estimates illustrate that reported ORB prevalence depends substantially on the availability and completeness of prospective trial records, study populations, outcome definitions, and the methodological criteria used to identify discrepancies [14,55].

Bias also occurs at the level of evidence synthesis. A 2025 meta-epidemiological study examining RCTs identified suspected ORB in 138 of 287 trials (48.08%). Statistically significant outcomes were more than three times as likely to be reported as nonsignificant outcomes [26]. Together, these findings show that ORBs may affect both the interpretation of individual RCTs and the reliability of the systematic reviews built from them, providing a strong rationale for rigorous prospective specification and transparent reporting of outcome changes.

Transparency, reporting standards, and prevention

Given the potential consequences of ORB for both individual trials and evidence synthesis, transparency safeguards are essential for preserving the interpretability of RCT findings. One of the most important safeguards is prospective trial registration. Prospective clinical-trial registration was introduced to create a public, time-stamped record of a study before its results were known. Trial registration permits subsequent comparison of the outcomes originally specified with those presented in the final publication, making discrepancies detectable [15,20,25,28,32]. However, registration itself cannot prevent selective reporting. Its effectiveness depends on timely registration, sufficiently detailed outcome descriptions, preservation of version histories, and systematic comparison of the final publication with the registered plan [25,45,56]. Empirical comparisons demonstrate why registration alone is insufficient. A 2014 observational study compared registered and published information for RCTs in the BMJ and JAMA and identified primary-outcome discrepancies in 18 of 39 (47%) registered BMJ trials and seven of 36 (19%) JAMA trials. The authors concluded that compulsory registration has limited value when registry information is incomplete, or discrepancies between registration and publication are not disclosed [56].

The usefulness of registration records also depends on the precision with which outcomes are prospectively described. Transparency requires more than naming an outcome; a sufficiently complete outcome definition should specify elements such as the measurement variable, assessment time point, analysis metric, method of aggregation, and method of assessment [21,50]. Incomplete prospective definitions may leave multiple possible outcome specifications compatible with the registered plan, thereby reducing the registry's ability to identify subsequent changes in outcome reporting [50]. This level of specificity reduces the number of plausible post hoc endpoint definitions and makes it possible for readers to determine whether the final analysis corresponds to the prospective plan [21]. Detailed protocols and statistical analysis plans can further strengthen this process by documenting the intended methodology in greater detail than is typically possible in a registry entry.

Transparency throughout the trial lifecycle is essential to mitigating ORB, particularly when prespecified methods or outcomes are modified. Established reporting guidelines promote accountability by requiring disclosure and justification of such changes [38,57,58]. Additional safeguards include prospective trial registration, sufficiently detailed prespecification of primary and secondary outcomes, public accessibility of study protocols and statistical analysis plans, and preservation of time-stamped amendments [18,21]. Any deviations from prespecified outcomes should be explicitly identified and justified in the final publication. Such changes can be documented through dated updates to national registries and public databases of clinical studies, such as ClinicalTrials.gov, or other trial registries, with revised protocols and explanations made available in journal supplementary appendices, ensuring that the nature, timing, and rationale of amendments remain accessible. Importantly, responsibility for ensuring transparency extends beyond trial investigators, as peer reviewers and journal editors should independently compare published outcomes against the original protocol and trial registry rather than assuming consistency [18,59].

The CONSORT statement provides standardized recommendations for reporting RCTs, enabling readers, reviewers, and journal editors to evaluate consistency between prospectively planned and published findings. The guidelines call for disclosure of important changes after trial commencement, including outcomes or analyses that were not prespecified, together with the reasons for those changes, as well as detailed descriptions of prespecified primary and secondary outcomes, including measurement variables, analysis metrics, methods of aggregation, and assessment time points [37,60]. Despite the availability of these standards, adherence remains inconsistent. A 2019 cross-sectional retrospective analysis of 39 randomized trials found that an average of 51% of CONSORT 2010 criteria were fulfilled; only one trial reported outcome changes after trial commencement with reasons, as required by the relevant checklist item. This finding illustrates that the availability of reporting standards does not necessarily ensure adequate disclosure of outcome modifications in practice [38].

Similarly, another retrospective analysis examined 72 randomized trials and found a mean CONSORT 2010 item fulfillment rate of 59%, with 22 trials (31%) fulfilling fewer than half of the assessed criteria [54]. Importantly, the existence of registration requirements and reporting guidelines does not itself ensure adherence. Despite longstanding preventive efforts, trials continue to change prespecified outcomes without adequate justification, demonstrating that safeguards can fail when deviations are not transparently disclosed or are not identified through comparison of the final publication with the prospective record [59]. Complementing reporting guidelines, the Cochrane Risk of Bias 2 (RoB 2) tool provides a structured framework for assessing potential bias in the results of RCTs. RoB 2 evaluates five domains of potential bias, including bias in the selection of the reported result, and assigns an overall judgment of low risk, some concerns, or high risk [61]. Whereas CONSORT encourages complete disclosure, RoB 2 enables reviewers to evaluate the risk that selective reporting has influenced an individual trial result.

Prevention also requires recognizing the limitations of registration when these additional safeguards are absent. Continuing problems with unregistered or late-registered trials demonstrate that stronger enforcement and editorial oversight remain necessary. This problem persists in contemporary trial literature. In a 2024 meta-epidemiological study of 361 randomized trials included in a large Cochrane review of exercise interventions for chronic low back pain, only 23.3% were prospectively registered. Registered trials had lower risk of bias and higher reporting quality than unregistered trials, while prospectively registered trials were more likely than retrospectively registered trials to have a low risk of reporting bias [62]. Approaches such as Registered Reports may further improve transparency by evaluating study questions and methods before results are known [29]. Taken together, these measures shift transparency from a single act of registration to a continuous process of prospective specification, documented amendment, independent comparison, and complete reporting throughout the trial and publication lifecycle.

## Conclusions

The persistence of ORB in randomized clinical trials highlights an important limitation in existing research transparency and reporting practices. Although prospective trial registration and established reporting guidelines have improved the ability to identify discrepancies between planned and published outcomes, these safeguards alone cannot ensure complete and unbiased reporting. When outcomes are selectively reported, treatment efficacy and safety may be misrepresented, with consequences extending beyond individual trials to systematic reviews, clinical practice guidelines, and evidence-based clinical decision-making. Inadequate transparency can obscure selective reporting and prevent independent assessment of the validity of trial findings. Addressing these limitations requires greater emphasis on detailed prospective outcome specification, the transparent documentation and preservation of original trial records, and systematic verification of outcome consistency during peer review. Strengthening these practices is essential to minimizing reporting bias, preserving the credibility of RCT findings, and ensuring that clinical decisions are informed by a complete, precise, and accurately represented evidence base.
